# The C-terminal region of *Trypanosoma cruzi* MASPs is antigenic and secreted via exovesicles

**DOI:** 10.1038/srep27293

**Published:** 2016-06-08

**Authors:** Luis Miguel De Pablos, Isabel María Díaz Lozano, Maria Isabel Jercic, Markela Quinzada, Maria José Giménez, Eva Calabuig, Ana Margarita Espino, Alejandro Gabriel Schijman, Inés Zulantay, Werner Apt, Antonio Osuna

**Affiliations:** 1 Departamento de Parasitología, Grupo de Bioquímica y Parasitología Molecular, Campus de Fuentenueva, Universidad de Granada, 18071 Granada, Spain; 2 Center for Immunology and Infection (CII), Biology Department, University of York, York, UK; 3 Parasitology Reference Laboratory, Instituto de Salud Pública de Chile, Avenida Marathon 1000, 7780050 Santiago, Chile; 4 Departamento de Microbiología y Parasitología, Facultad de Medicina, Universidad de Panamá, República de Panamá; 5 Servicio de Microbiología Hospital Universitario y Politécnico La Fe, Valencia, Spain; 6 Laboratory of Immunology and Molecular Parasitology, Department of Microbiology, University of Puerto Rico, School of Medicine. PO BOX 365067, San Juan, 00936-5067, Puerto Rico; 7 Laboratorio de Biología Molecular de la Enfermedad de Chagas, INGEBI-CONICET, Buenos Aires, Argentina; 8 Laboratorio de Parasitología Básico-Clínico, Instituto de Ciencias Biomédicas, Facultad de Medicina, Universidad de Chile, Santiago, Chile

## Abstract

*Trypanosoma cruzi* is the etiological agent of Chagas disease, a neglected and emerging tropical disease, endemic to South America and present in non-endemic regions due to human migration. The MASP multigene family is specific to *T. cruzi*, accounting for 6% of the parasite’s genome and plays a key role in immune evasion. A common feature of MASPs is the presence of two conserved regions: an N-terminal region codifying for signal peptide and a C-terminal (C-term) region, which potentially acts as GPI-addition signal peptide. Our aim was the analysis of the presence of an immune response against the MASP C-term region. We found that this region is highly conserved, released via exovesicles (EVs) and has an associated immune response as revealed by epitope affinity mapping, IFA and inhibition of the complement lysis assays. We also demonstrate the presence of a fast IgM response in Balb/c mice infected with *T. cruzi*. Our results reveal the presence of non-canonical secreted peptides in EVs, which can subsequently be exposed to the immune system with a potential role in evading immune system targets in the parasite.

Chagas disease is caused by *Trypanosoma cruzi*, a flagellated protozoan belonging to the Kinetoplastid order, which affects to approximately 7.5 to 10 million people, causing approximately 10,000 deaths every year^1^,^2^. This disease is a major public concern in endemic areas of Central and South America, but cases can be also found in non-endemic countries due to globalization and migratory movements from endemic areas^3^. The course of the disease is marked by two phases: an acute phase with a mortality rate of some 5%, and a chronic phase 5 to 8 weeks after the onset of the illness, which remains asymptomatic for a long time; in fact, only 30% of cases ever develop any symptoms related to the disease^4^. The acute phase of the infection in murine experimental models gives rise to an enhanced polyclonal humoral response, which is responsible for dysregulating the immune response and plays an important part in establishing the parasite within the host in preparation for the chronic phase^5^,^6^. This hypergammaglobulinemia appears two weeks after the onset of the disease and increases during the following two weeks until it reaches its highest level of its specific antibody response, which then persists throughout the beginning of the chronic phase (after 60 days), with a rise in IgM, IgG1, IgG3 and IgG2b and a predominance of the IgG2a isotype, which increases to ten times its normal value^7^,^8^.

The activation of B cells that comes into play in the course of infection is due to the linear or conformational epitopes contained in an antigen, which are capable of giving rise to a humoral response owing to their tendency to be exposed to the immune system, their molar ratio and/or their particular conformational organization^9^,^10^. The high concentration and affinity of antibodies against a particular antigen may also be due to the existence of genes that encode proteins with repeated domains, which are defined as possessing one or more copies of an amino-acid pattern^9^,^10^. This immunodominance is responsible for the response of B lymphocytes to parasites such as *Plasmodium falciparum*^11^,^12^, *Leishmania infantum*^13^,^14^ and *T. cruzi*^10^,^15^,^16^.

Some of the most immunogenic epitopes discovered in *T. cruzi* are composed of repeated sequences (RSs) or sequences repeated in tandem (TRSs)^10^. These RSs represent a large number of the total number of genes belonging to *T. cruzi* and correspond mainly to its three great gene families: *trans*-sialidases (TSs), mucins and mucin-associated surface proteins (MASPs)^17^,^18^. Antigens such as SAPA (Shed Acute Phase Antigen), sequences of the conserved C-terminal of TSs or sequences of the central TSSA (Trypomastigote Small Surface Antigen) region, belonging to the TcMUCIII mucin family, have been characterized in the TS and mucin families^8^,^9^,^10^,^11^,^12^,^13^,^14^,^15^,^16^,^17^,^18^,^19^,^20^,^21^.

Several recent works have demonstrated the presence of TS, mucin and MASP proteins as part of the protein composition of Exovesicles (EVs) secreted by *T. cruzi*^23^,^24^,^25^,^26^,^27^,^28^. EVs are defined as membrane-bound particles enclosed by a lipid bilayer and released by cells into extracellular environment^27^. Depending on the origin, EVs in *T. cruzi* have been classified into two types, the first type are exosome-like EVs (mean size ~70–90 nm), originating from multivesicular bodies (MVBs) and found to be associated with the flagellar pocket, a structure related with the endocytic and exocytic pathways in kinetoplastid parasites; the second type are ectosome-like EVs, which are bigger sized structures (mean size ~130–140 nm) that are released after the fusion of multivesicular bodies (MVBs) with the plasma membrane^27^. EVs are known to play a major role in intercellular communication and the immune response to parasites^27^. For instance, the injection of shed vesicles increases the mortality in mice infected with *T. cruzi* due to the production of proinflammatory cytokines, thus playing a pivotal role in the pathogenesis of Chagas disease^29^.

A peptide array screening using up to 110 MASP peptides has confirmed the presence of a humoral response against hypervariable regions of these proteins in acutely infected mice^30^. However, the response against conserved MASP regions has not been investigated so far. The high levels of expression during the infective stages, conservation of the C-terminal (C-term) region of MASPs and its presence in exovesicles render this region susceptible to be targeted by the immune system, as revealed by the results presented in this study.

## Materials and Methods

### Human serum samples

All protocols involving human subjects were approved by the Research Ethics Committee of the Institute of Public Health and Faculty of Medicine University of Chile, the Gorgas Commemorative Institute for Public Health in Panama and La Fe Hospital (Valencia) and University of Granada in Spain. All study participants provided written informed consent prior to initiation of study activities and the experiments were performed in accordance with the approved guidelines.

The pool of positive sera came from chronic chagasic patients, while negative sera came from healthy controls from the Gorgas Commemorative Institute for Public Health in Panama (n = 26). The sera from chagasic patients were serum-positive as confirmed by the following immunological test: CHAGAS ELISA IgG + IgM (Vircell) and CelQuest Chagas ELISA (ATGen Diagnóstica) and by PCR. Sera from healthy Chilean and Panamanian subjects (n = 33) that had proved to be negative with the same analyses were used as a pool of negative controls. The sera from the La Fe Hospital (Valencia, Spain) (n = 15) corresponded to an immigrant population from different countries, diagnosed also by at least two commercially available immunological methods and confirmed by PCR and direct microscopic examination of blood smears. These patients were classified according to their symptoms being gastrointestinal, cardiopathic and asymptomatic respectively.

### Parasites

Epimastigote forms of the CL- Brener (DTU TcVI) and PAN4 (DTU TcI) stocks of *T. cruzi*^31^ were cultured at 28 °C in MTL medium supplemented with 10% heat-inactivated fetal calf serum (IFCS) as described by Ruiz-Perez *et al.*^32^. Infective trypomastigote metacyclic forms (M) (70–80% of the cultivated forms after 9 days’ culture) were obtained *in vitro* in modified Grace’s medium and purified by centrifugation in Percoll (GE healthcare) as described by Castanys *et al.*^33^.

### Cloning and sequencing of the MASP C-term region

Total RNA from M Pan4 and CL-Brener strains was purified using the RNeasy Mini kit (Qiagen, Hilden, Germany). Complementary DNA (cDNA) was synthesized using random primers and the iScript cDNA synthesis kit (Bio-Rad Laboratories, Inc). To obtain the different sequences corresponding to the conserved C-term region, the MASP-C F 5′-GGTCTCCCACACCACCTC-3′ and MASP C R 5′-CCACCACCGCAGTAGCAG-3′ primers were used. All the amplification products were purified and cloned in pGEM-T^®^ Easy Vector (Promega) before being sequenced and analyzed. To test the quality of our cDNA samples, total RNA samples were also amplified with the primers described above.

### Synthetic peptides and bioinformatic analysis

To obtain the consensus sequence of MASP C-term region of 200 sequences from *T. cruzi* genome data base (http://tritrypdb.org/tritrypdb/) we used the Multiple Em for Motif Elicitation 4.3.0. (MEME) program (http://meme-suite.org/tools/meme). The analysis was done with the maximum number of characters ( = 200 MASP C-term sequences) permitted by the program as input. Comparative analysis between the sequenced clones was undertaken using the ClustalW program (http://www.ebi.ac.uk/Tools/msa/clustalw2/) and the prediction of GPI anchor sites with big-PI program (http://mendel.imp.ac.at/sat/gpi/gpi_server.html). The obtained C-term consensus sequence was synthesized for a total of six overlapping peptides in 12 of their 15 amino-acids, which we refer to as C1, C2, C3, C4, C5 and C6, together with a peptide called C7, corresponding to the C-terminal end of the MASP52 protein, which has amino-acid substitutions in three of its residues^34^. All the peptides were synthesized by GenScript to >98% purity (USA, Inc).

### Infection of Balb/c mice

Animal experiments were performed in accordance with institutional guidelines (Spanish government regulations (Real Decreto RD1201/05)) and European Union guidelines (European Directive 2010/63/EU) and were approved by the local authorities (Resolution n° 522 of the Ethics Committee of the University of Granada). Three groups of 5 four-week-old, female, Balb/c mice were infected with 1 × 10^5^ metacyclic trypomastigote forms of *T. cruzi*, the PAN4 strain, resuspended in PBS and administered by IP injection in order to assess the humoral response at different times post-infection (pi). After 3 days we proceeded to evaluate parasitemia of the trypomastigote forms present in the blood stream by studying smears from 15 μl blood samples taken from the mice by bleeding a section of the tail vein, after fixing with methanol and staining with Giemsa.

To obtain serum during the acute phase we bled three of the mice by cardiac puncture on weeks 2, 3 and 4 pi; remaining mice were bled after 20 weeks, during the chronic phase of the illness. The sera were centrifuged at 300 g for 30 min divided into aliquots, and stored at −20 °C until use.

### ELISA

For the ELISA assays, each well of 96-well Nunc Immobilizer^TM^ microtitration plates (Thermo scientific) was coated with 1 μg of one of the peptides diluted in Bicarbonate/carbonate coating buffer (100 mM) pH 9.6. The plates were then washed 3 times with PBS-Tween 0.2% (PBS-T) and blocked with skim milk at 2% in PBS-T for 1 h at 37 °C. They were subsequently washed 3 times with PBS-T and incubated for 45 min at 37 °C with 100 μl of a 1:200 solution of primary antibody diluted in PBS. Thereafter, the plates were washed 3× with PBS-T and then incubated for 30 min at 37 °C with a 1:1000 solution of secondary anti-human IgG antibody (Sigma-Aldrich) and/or anti-mouse IgG and IgM (Sigma-Aldrich), all horseradish peroxidase-labeled in 100 μl of PBS. After incubation with the secondary antibodies, the plates were washed 4× and O-phenyl-diaminobenzidine plus 30% H_2_O_2_ (1 μl/ml) (Sigma-Aldrich) was added to 0.05 M phosphate-citrate buffer, pH 5.0 as a peroxidase substrate before further incubation for 15 min at 27 °C. The reaction was stopped with a solution of 0.1 M 2 N H_2_SO_4,_ and absorption measured at 492 nm in an ELISA Multiskan spectrum reader (Thermo). Sera from healthy individuals living in the same areas of Chile and Panama or mouse preinfected sera were used to calculate the cut-off value using the formula: median + 3 × SD of the negative samples.

### Avidity ELISA and epitope mapping

To perform the epitope mapping assay and remove low-affinity antibodies, KSCN was used as chaotropic agent to increase the stringency of the assay. Briefly, after primary antibody incubation and washing 3× with PBS-T, each well was incubated with 0.5 M KSCN for 15 min at 37 °C. Then the wells were washed 3 times with PBS-T, following the aforementioned further steps. We calculated the % avidity of the pool of positive sera, ((mean OD of KSCN-treated sera/mean OD KSCN-untreated sera) × 100%). Indexes above 40% were considered of high avidity^30^,^35^.

### Isolation of IgGs against the C7 peptide and immunofluorescence assays

IgGs from pooled human positive and negative sera were purified according to the manufacturer’s instructions using the protein A HP spin trap kit (GE Healthcare).

To purify the specific IgG fraction against the C7 peptide, nitrocellulose strips were coated with the C7 peptide and then immune adsorbed with the IgG purified as described above. The adsorbed antibodies were eluted with buffer glycine-HCl 0.1 M (pH 3.5), restoring the neutral pH of the solution by adding 1 M Tris-HCl buffer (pH 8.0) supplemented with 0.1% purified bovine serum. The purified IgGs were stored in aliquots at −80 °C until use.

To determine the effectiveness of the process, the eluted IgG fractions were tested by ELISA using Microtiter plates (Nunc, Denmark) coated with the C7 peptide (10 μg/ml) with a titer of at least 1/6400.

Confocal microscopy was performed by using epimastigote forms cultured at the end of the logarithmic phase. The parasites were fixed with paraformaldehyde at 3.7% for 15 min at room temperature, washed 3× with 0.02 M Glycine in PBS, and then permeabilized with 10 mM citric acid, 100 ml H_2_O, 100 μl NP-40, pH 6 for 5 min at 95 °C. After permeabilization step, the samples were incubated with a 1:500 dilution of either positive and negative IgGs purified as described above, and the remaining steps were as previously described^36^.

### Cr^51^ labeled epimastigote forms

The complement lysis assays were carried out in sterile glass tubes in which a suspension of the parasite forms was adjusted to 1 × 10^7^ epimastigote forms/ml, the medium was eliminated by centrifugation at 1500 g for 15 min and replaced with RPMI (Gibco LTD. Middlesex, England; UK) medium with 10% inactivated fetal bovine serum plus 50 mCi/ml of Cr^51^ (specific activity of 200–900 Ci/mmol) (DUPONT, USA).

After incubating for a 2 h, at 28 °C, the parasites were washed by centrifugation, and resuspended in cold nonradioactive medium, and the parasite pellet resuspended in Veronal Buffer (VB, 10 mM barbital/NaCl 145 mM, CaCl 0.15 mM/MgCl 0.5 mM, pH 7.4) (Virion).

To calculate the CH_50_ (50% cell lysis by complement (CHS)) the ^51^Cr labeled epimastigote forms (2.5 × 10^6^ parasite/ml) were treated with different dilutions of Complement (CHS) (Sigma-Aldrich) (1:200, 1:100, 1:50 and 1:25) in Veronal Buffer plus 10% of heat-inactivated (56 °C 30 min) Chagas positive (1:500 by ELISA) human pool sera and incubated at 37 °C 1 h. Thereafter, the different tubes were centrifuged at 16000 g for 5 min. The supernatant was transferred to medium tubes. Each parasite-containing pellet was treated with 1 ml of SDS 0.1%-NaOH 1 N and transferred to different measurement tubes. The different samples were suspended in Ultima Gold LC Cocktail (Sigma) liquid scintillation medium and the radioactivity of the pellet as well as supernatant were determined by a γ-spectrometer (Beckman, LS6000) to calculate the specific release of ^51^Cr, using the following formulae: %LE = (CPMSbn.ctrl/CPSMSbn.Ctrl + CPMpelletCtrl) × 100; %LSP = (CPMSbn.Prb/CPSMSbn.Prb + CPMpelletPrb) × 100. Where: %LE = spontaneous liberation; %LSP = specific liberation; CPMSbn = CPM in supernatant; CPM pellet = CPM in the pellet; Ctrl = control; Prb = problem.

The calculation of the complement DL50 parasite lysis (50 CHS) was obtained by lineal regression with the specific release data of the different Complement dilution with the Graph Path InStat version 3.06 software.

### Complement inhibition by MASP C-term peptides

After the determination of the 50 CHS, the labeled E -^51^Cr forms were incubated with 10% of heat- inactivated, different pool of positive human sera from Chagas disease patients (1:1500), verified by ELISA, with three different pathologies: gastrointestinal, cardiopathic or asymptomatic supplemented with the optimal concentration of CHS (CH50 = 1:18) and 1 μg/100 μl of each peptide (C1–C7) at 37 °C for 1 h. As a positive control, we used the pooled sera of Chagas disease patients without inactivation, and as negative control the pool of inactivated serum without the additional complement.

At the end of the incubation, the samples were processed and measured as described above. The data of the percentage of lysis inhibition was calculated by the formula %IC = (LspC − Lsp/Lsc) × 100, where %IC is the percentage of complement inhibition.

### EVs purification

For the isolation and purification of EVs from bloodstream tripomastigotes, 5 × 10^7^ parasites were incubated for 12 h in free EVs medium (RPMI 1640 supplemented with 10% IFCS, which had previously been centrifuged at 100000 g for 1 h). Next, the parasites were centrifuged at 3000 g for 15 min to eliminate remaining of cells and the supernatants were submitted to a protocol of differential centrifugation as previously described^37^. In brief, cell-free media were centrifuged at 17000 g for 20 min, and the supernatants filtered in a 0.2 μm filter (Sartorius Minisart) to eliminate any cell debris. The filtered media were finally ultra-centrifuged at 100000 g for 1 h. The pellets were re-suspended in PBS, washed 3× in PBS and ultra-centrifuged again at 100000 g for 1 h. The pellets from the second ultra-centrifugation were re-suspended in PBS and stored at −20 °C until used. All steps were performed at 4 °C in an ultracentrifuge Avanti^®^ J-30I (Beckman Coulter) with a JA-30.50 Ti rotor.

To confirm the presence of exosomes in our samples, the pellet was re-suspended in 0.1 M Tris-HCl pH 7.2 after the last centrifugation. A drop of the suspension was placed on the surface of a Formvar/Carbon copper grid and stained by negative stain with 2% v/v uranyl acetate for direct observation by transmission electron microscopy. The diameter of EVs was measured by ImajeJ 1.41 software or by light scattering using the Zetasizer Nano range system (Malvern Instruments).

### Immuno Electron Microscopy (EM)

The EVs obtained as described above were fixed with Karnovsky’s fixative^38^ (2.5% (v/v) glutaraldehyde, 2%(v/v) formaldehyde in PBS 1X) 4 h at 4 °C and then dehydrated and embedded in LR White resin (Sigma). The ultrathin section on grids was blocked in a solution containing 1% (w/v) gelatine from cold-water fish skin (SIGMA) prepared in PBS 10 min RT, then, placed on three drops of 0.02 M glycine in PBS for 5 min each of them, and finally blocked with 1% albumin from chicken egg white (Sigma) for 15 min at RT. The grids were placed in a wet chamber and incubated with 20 μl of a 1:20 dilution of anti C-term MASP and anti-clathrin (SCBT) antibodies for 90 min at RT. To eliminate the unbound antibodies, the grids were washed 5× in PBS for 25 min and then immersed in a 1:20 dilution of anti-rat and anti-rabbit polyclonal antibodies labeled with 10 and 25 nm gold-particles, respectively. Afterwards, the grids were washed 4× (5 min each) in PBS followed by washing step of 5 min in distilled water. The samples were finally contrasted with 2% (v/v) uranyl acetate solution and the EM observations were carried out with a Zeiss (Libra- 2000) transmission microscope.

### Statistical analysis

To analyze the humoral response against the peptides in the mice samples we first determined the cut-off value via the mean value of the negative controls ±3× SEM. All statistical calculations were made with GraphPad Instat v 3.05 software. For all tests, a P value of <0.05 was considered significant. The data of the absorbance were represented with the Slide Write plus for Windows V 7.0.

## Results

### MASP amino-acid sequence analysis

**Figure 1:**
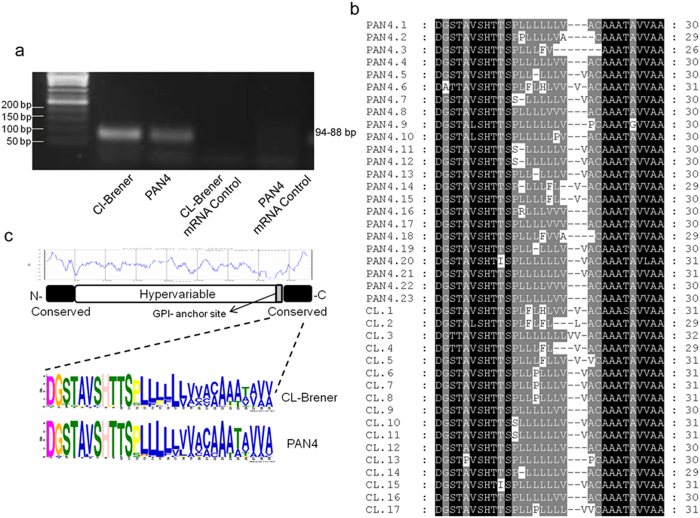
Analysis of the expressed MASP C-terminal amino-acid sequences. (**a**) Agarose gel (2%) of the amplicons of the MASP C-terminal region (94-88 bp) using cDNA from the CL-Brener and PAN4 strains as a sample and including an mRNA quality control for both strains. (**b)** Multiple alignment of the sequences obtained from the C-terminal region expressed in the PAN4 (23 clones) and CL-Brener (17 clones) strains. Residues showing 100% conservation are in black and those showing >80% are in grey. (**c**) The graph (blue lines) represents a Kyte-Doolitle hydrophaty plot of a prototypical MASP sequence schematized below it. The amino-acid frequencies of the C-terminal end of the MASPs expressed by both strains were elucidated by MEME (see material and methods) and are represented at the bottom.

To assess possible differences at the amino-acid level, we amplified the MASP C-term region of CL Brener (TcVI) and PAN4 (TcI), two phylogenetically divergent strains of *T. cruzi* isolated in Brazil and Panama, respectively. We sequenced a total of 17 clones of the CL-Brener strain and 23 of PAN4 (Supplementary Fig. S1). When analyzed, and similarly to what was observed in *T. cruzi* mucins^20^, the predicted putative GPI anchor attachment (ω-site) site of the C-term region was aspartic acid (D, aa residue 1) in 30/40 sequences, while serine was present (S, residue 3) in 8/40 and glycine (G, residue 2) in 1/40 sequences. All sequences showed a high degree of homology from residues 1–11 and from aa 20 to 26–31, with a less conserved hydrophobic region from aa 12–20 (Fig. 1a). However, substitutions were mostly between aa with a neutral charge, thus maintaining the characteristic hydrophobicity of this region (aa in blue, Fig. 1c). It can be concluded that the C-term region was very similar at the amino-acid level and its physico-chemical characteristics highly conserved in all the clones analyzed.

### MASP C-term region localization in exovesicles

**Figure 2:**
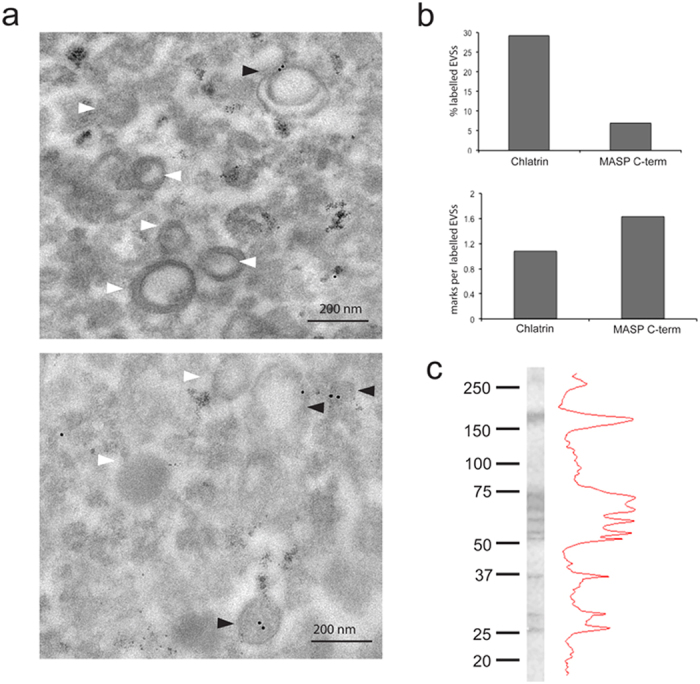
Trypomastigote Exovesicles (EVs) cargo MASP C-term region. **(a)** Immunogold labeling using antibodies against the MASP C-term region. White arrows: Non labeled EVs. Black arrows : labeled EVs (**b**). Quantification of EVs labelling. Bar graphs are representative of at least 100 EVs. (**c)**. Western blot of protein extracted from EVs using antibodies against MASP C-term region.

Electron microscopic analysis of isolated EVs of human infective trypomastigote forms revealed a spherical shape, some as cup-shaped membrane encapsulated particles with a size varying from ~90 to 300 nm with a mean of 154.30 nm ± 7.58 nm (although differences may correspond to the sagittal cut of the samples). Using immunogold labeling, specific antibodies against the MASP C-terminal region showed the presence of MASP proteins bearing the C-term peptide on the EVs (Fig. 2a). After analyzing all EVs, the number of EVs containing the MASP C-term region was ~7% with a mean/average of 1.6 ± 0.8 marks per EVs, whereas clathrin (EVs putative marker) was located in ~30% of the EV population having 1± 0.2 marks per EV (Fig. 2b). The fact that MASP proteins were located in a subpopulation of EVs reflects the different protein cargo of the secreted particles by the parasites. To further confirm the presence of MASP proteins bearing the C-term in EVs, we performed western blots using antibodies against this conserved region in samples of trypomastigote EVs. The size pattern of proteins recognized ranged from ~75 to 25 kDa (with the exception of a band of ~170 kDa), which is within the predicted size range for proteins belonging to the MASP family.

### Humoral response to the C-term region of MASP proteins

**Figure 3:**
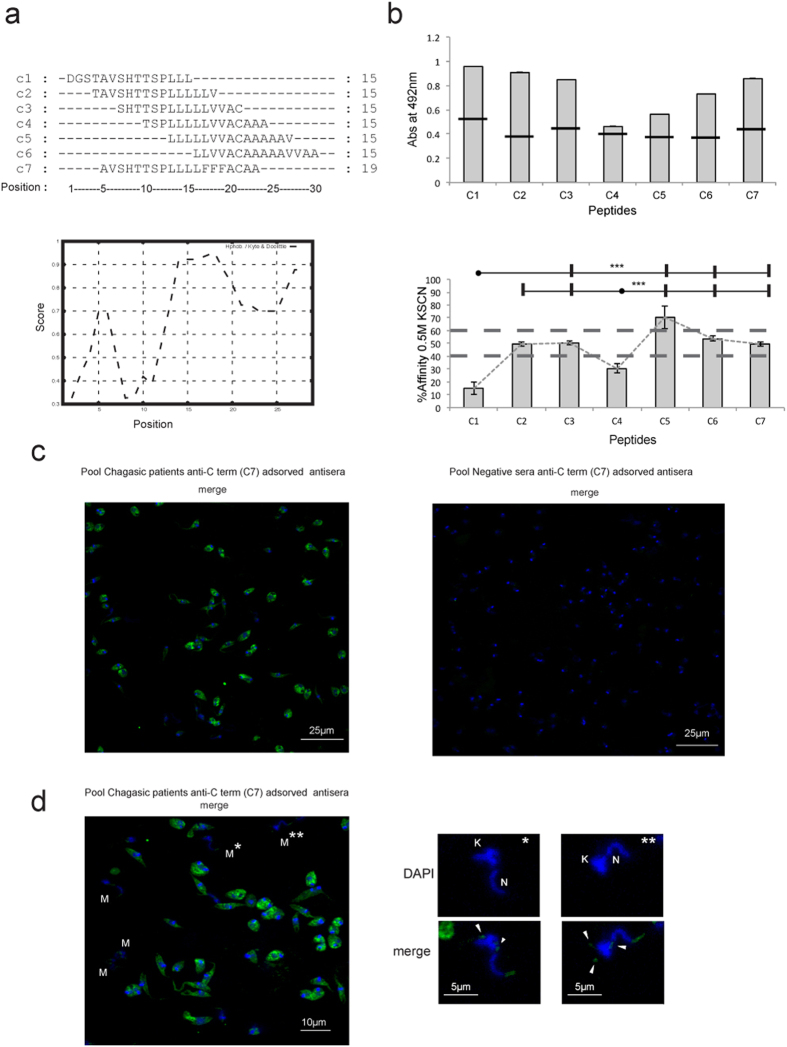
Mapping the affinity of the epitopes in the C-term region of MASP proteins. (**a**) Sequence of overlapping amino acids used in this study and Kyte-Doolitle hydrophaty plot (bottom graph) of the consensus C-term MASP region (**b**). Mapping of the affinity of the epitopes for a pool of sera from patients suffering from chronic Chagas disease. The cut-off value (top graph, tranversal black lines) was calculated using a pool of negative sera from Panama using the following formula: Mean + 3 × SD pool of negative samples. Peptides with affinity levels (dashed lines) >40% and <60% were considered of medium affinity and >60% of high affinity. P values of <0.001 (***) (bottom graph) were considered as extremely significant. (**c**) IFA assay using a Pool of positive (left) or negative (control, right) sera adsorbed against the C7 peptide. (**d**) Location of the MASP C-term region in selected metacyclic trypomastigotes (labeled as “M”). “N” indicates the nucleus and “K” the kinetoplast. The white arrows represent the location of MASP C-term in vesicles.

Having demonstrated the presence of MASP C-term in EVs, the associated humoral response to this region was investigated in different samples of chagasic patients. We first searched for possible differences in the recognition of this region (30 aa long) by synthesizing 6 overlapping peptides (C1–C6) corresponding to the consensus sequence of the MASP C-term region. Besides, we also analyzed the C-term region of the MASP52 protein (peptide C7), which we had characterized in a previous study^34^ and found to have three substitutions in the consensus sequence: Leu-Leu-Val instead of Phe-Phe-Phe at positions 16–18. Hence, peptide C7 served as a control to ascertain whether amino-acid substitutions with the same polarity entailed changes in the humoral response (Fig. 3a).

Tests on the 7 peptides against a pool of sera from patients with chronic Chagas disease proved positive for all the peptides (Fig. 3b). In order to rule out low affinity antibodies, we characterized the affinity of the antibodies in the same sera for the different peptides. Peptide C5 showed an affinity of over 60%, while C2, C3 and C7 affinities fell within 40–60%, and C1 and C4 were the peptides with lowest affinities (below 40%) (Fig. 3b, bottom panel). These results shown that most of the C-term region has specific antibody recognition. It should be kept in mind that protein C7, which has three amino-acid substitutions for amino acids with the same charge, maintains an associated humoral response.

To confirm the presence of a humoral response not only against linear synthetic peptides but also to conformational epitopes in the MASP C-term region in *T. cruzi*, we performed an IFA assay using the IgG fraction of pooled negative and positive sera (the same as above) adsorbed against the C7 peptide. The IFA assay showed that the positive-pool sample contained antibodies highly specific to the C7 peptide, not cross-reacting with the negative control (Fig. 3c). Nevertheless, it has to be kept in mind that the antibodies that recognize peptide C7 in the positive sample may also recognize any other MASP proteins due to high levels of homology of this peptide with other members of the family. Hence, the recognition pattern would probably be due to a set of MASP proteins rather than an individual protein. It is noteworthy noting that the MASP C-term region was mainly located in small rounded foci close to the kinetoplast in metacyclic trypomastigotes (Fig. 3d), which is in accordance with the presence of this region in EVs (Fig. 2a).

**Figure 4:**
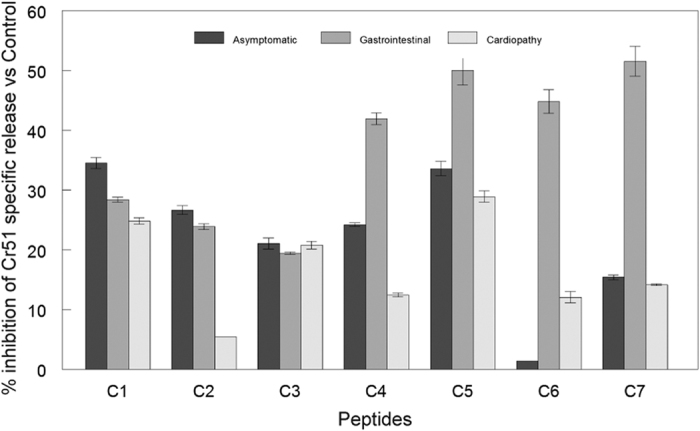
Complement inhibition by MASP C-terminal peptides. Cr^51^ labeled Epimastigote forms were treated with 10% heat inactivated sera from chagasic patients diagnosed as gastrointestinal, cardiopathic or asymptomatic. After incubation with 1/18 dilution human complement (CH_50_) and 1 μg/100 μl of each peptide (C1–C7) at 37 °C for 1 h. Pooled sera from chagasic patients without inactivation was used as positive control and a pool of inactivate sera without the supplement of complement as a negative control.The data percentage of Cr^51^ specific release was calculated by the formula %IC = (LspC − Lsp/Lsc) × 100, where %IC is the percentage of complement inhibition, Lsp the specific liberation of samples, LspC the specific liberation of positive control samples and Lspc the specific liberation on negative control samples.

**Figure 5:**
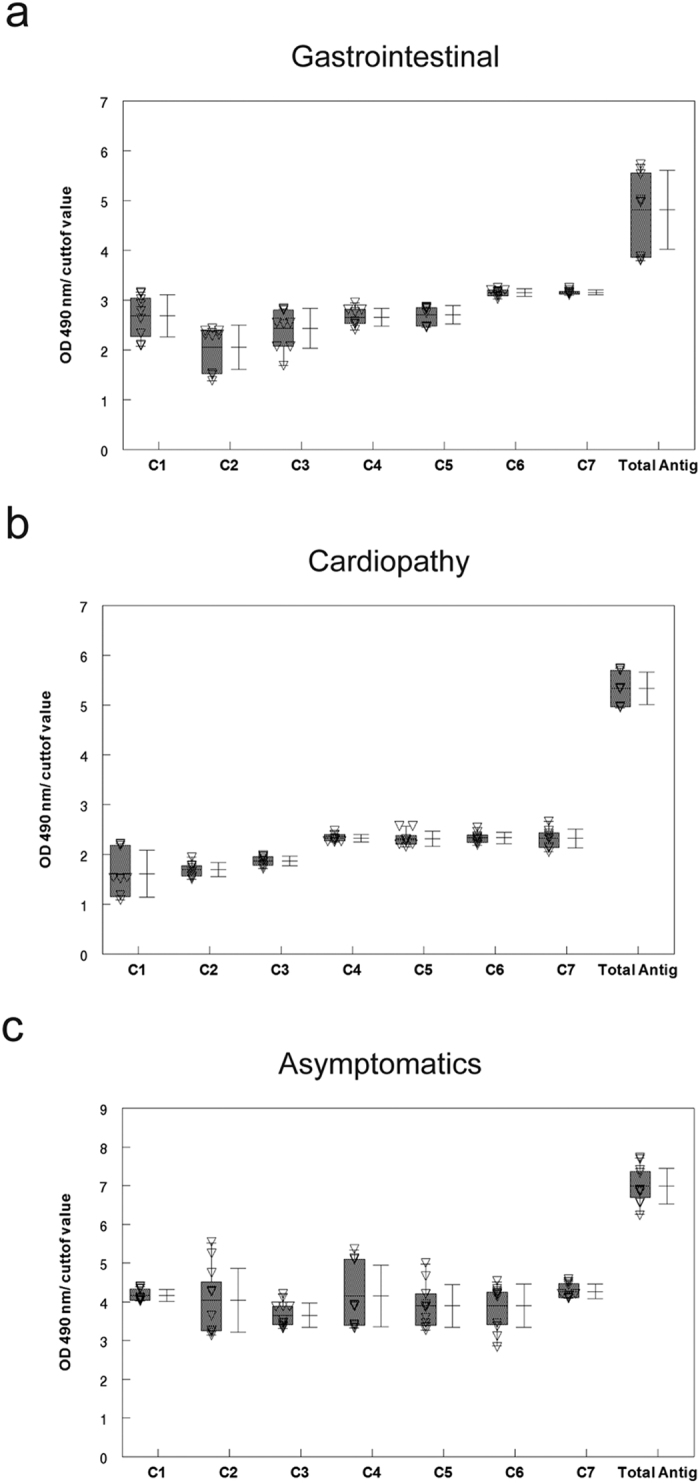
ELISA of peptides C1–C7 and parasite lysates (total antigen) using sera from patients infected with *T. cruzi* with different manifestations of the disease (gastrointestinal (**a**), cardiopathic (**b**) and asymptomatic (**c**)). All the samples with values for OD 492 nm sample/cut-off above 1 were considered positive. The cut-off value was calculated using a pool of negative sera from Panama with the following formula: Mean + 3 × SD pool of negative samples.

We also used the panel of peptides to test the inhibition of the complement activity primed by the presence of specific lytic antibodies against the MASP C-term region in pools of sera from patients with different pathologies (gastrointestinal, cardiopathic or asymptomatic). The Ca^2+^ independent inhibition is due to the ability to sequester specific antibodies and compete with antigens present on the surface of labeled epimastigotes, where the complement acts. All in all, we found an inhibition of the lytic activity for most of the peptides and sera, with an increased action for the sera from patients with gastrointestinal pathologies and a decrease for sera from asymptomatic patients (Fig. 4). Similarly to the affinity assay, the peptide C5 showed the highest percentages of inhibition, with the exception of gastrointestinal sera in which C7 was the most effective peptide blocking the action of lytic antibodies (Fig. 4). When the individual antibody response for the same sera was tested against the peptides, all of them were positive (Fig. 5). However, the values obtained for the peptides were lower compared to the control using total lysates of the parasites, which indicates that the response to the C-term region of is just a fraction of the total *T. cruzi* humoral response.

### Humoral response to the C-term region of MASP proteins in infected mice

**Figure 6:**
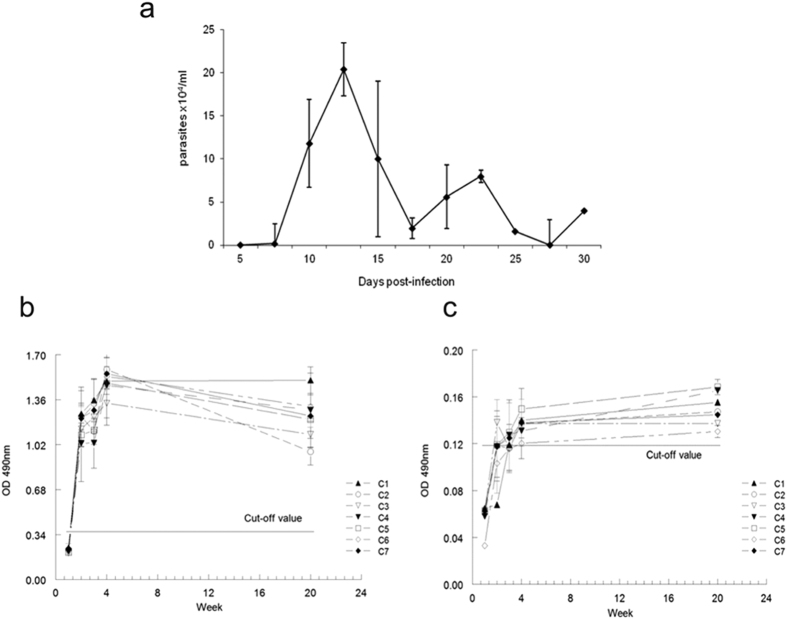
IgG and IgM humoral response against peptides C1–C7 in Balb/c mice infected with *T. cruzi*. (**a**) Parasitemia in blood was measured from day 5 to 30 pi (n = 3 mice per measurement) (**b**) Specific IgM response to the MASP C-terminal region against peptides C1 to C7; (**c**) Specific IgG response of the MASP C-terminal region against peptides C1 to C7. The cut-off values were obtained from the mean values of negative controls (sera from pre-immune mice) + 3 × SEM.

On determining the level of parasitemia in the blood of Balb/c mice infected with *T. cruzi* (CL-Brener), two distinct peaks for parasitemia during the course of the acute phase were obtained on days 12 and 22 pi, which is typical of infection with this strain (Fig. 6a). Parasitemia diminished subsequently until becoming imperceptible after the day 30 onwards.

When we analyzed the IgM response, we observed an early positive response from week 2 pi, which continued to be high until 20 weeks pi for all the peptides assayed (Fig. 6b). On the other hand, the IgG response only showed a positive signal for all peptides after 20 weeks pi (Fig. 6c), the titers against IgG always being lower than those in response to IgM.

The humoral response observed was not responsible for any significant differences among peptides C1 to C6, which make up the C-term consensus sequence, or with regard to C7, which, as mentioned above, contains three amino-acid substitutions, although the highest IgG response found was for the C5 peptide.

## Discussion

Much of the genome of *T. cruzi* is composed of repeated elements, among which the following multigene surface families are found: TS, mucin, MASP, dispersed gene family-1, gp63, TcTSAV or TcSMP^18^,^39^,^40^,^41^. The antigenicity of proteins belonging to the TS and mucin families has been previously described, and recent reports showed that peptides from the hypervariable MASP region could elicit IgM and IgG responses in experimental infections^30^. This, together with the increase in its expression during the infective forms of the life cycle of the parasite, the high number of transcripts (1.8–2.8% of the cell total) found in the trypomastigote form, and the location of many of its members in the outside of the parasite membrane^34^, make MASP proteins a likely target for the mammal host’s immune system, as has recently been demonstrated^30^. Due to the absence of any defined pattern in the hypervariable region of these proteins, we decided to analyze the humoral response against the C-term region of the MASP family, a commonly repeated motif that potentially serves as a signal for the addition of the GPI anchor at the endoplasmic reticulum.

Our analysis on the possible amino-acid variations in the C-term region confirms the high degree of conservation of this sequence (Fig. 1), indicating that this region may be extremely important for the functionality of the MASP family. It has been suggested that, at nucleotide level, it could be the site for homologous recombination^42^, or that it could direct the addition of the preformed GPI-anchor within the lumen of the endoplasmic reticulum at the protein level^43^.

Given that this MASP protein region is predicted to be cleavage for the addition of the GPI lipid moiety (potentially not present in mature proteins), we looked for alternative pathways for its exposure to the immune system. Recent findings have demonstrated that a large proportion of a parasite secretome is released through EVs, with the *T. cruzi* EV protein composition having been recently described^23^,^27^. Our results showed that the C-term region is present in a subpopulation of trypomastigote EVs (Figs 2 and 3d), also suggesting that the final exposure to the immune system of the MASP C-term region might be mediated via EVs. In this sense, two possible scenarios can be outlined: (i) a novel role of GPI signal sequences as transmembrane regions in lipid membranes. As mentioned above, the vast majority of mature GPI-anchored proteins lacks its C-terminal hydrophobic segment. However, there are examples of this region not being excised and instead providing a transmembrane region for mature proteins in human plasma membrane cells, like the Bone Marrow Stromal Cell Antigen 2 (BST-2)^44^. Thus, it is possible that MASP GPI signal sequences function as transmembrane regions in EVs membranes; (ii) release of misfolded proteins via EVs. In this case, the high production of GPI-anchored proteins in *T. cruzi* might lead to the generation of toxic by-products retaining the GPI-anchor signal peptide, that has not overcome ER quality control^45^. Without its native fold, the proteins would be loaded into MVBs, being finally released through EVs^46^. This mechanism of cellular homeostasis has been found in neurodegenerative diseases such as Alzheimer’s, Huntington’s, Parkinson’s and prion diseases and might be part of the regular functioning of *T. cruzi* cells and/or in response to stresses which would lead to the formation of misfolded proteins^45^,^46^.

Together with MASP proteins, recent proteomic analysis of *T. cruzi* EVs revealed the presence of proteins without any canonical secretory signals and diverse functionalities, with the most striking examples in proteins related with translation and nucleic acid binding^23^, such as the ribosomal protein P0, which is also recognized by serum of chagasic patients^47^. In the case of the C-term MASP region, our results using sera from chagasic patients revealed the presence of a humoral response against the different overlapping peptides, maintaining 5 out of 7 peptide affinities above 40% when low affinity antibodies were removed (Fig. 3c). Moreover, the IFA assay against the C7 peptide demonstrated that this response is also present for conformational epitopes.

The sequencing of the different isolated C-term clones and western blot analysis of trypomastigote EVs using anti-MASP C-term antibodies revealed the simultaneous co-expression of several members of this family in EVs, which is in agreement with the high diversity of expression of MASP proteins in *T. cruzi*^48^. Thus, we can conclude that the humoral response to the C-term region is not due to the expression of individual MASP members but to the simultaneous expression of numerous MASP proteins, which in turn allows presumption that the molar ratio of the antigen exposed to the immune system is very high.

We also confirmed the presence of an antibody response against the MASP C-term region in chagasic patients with different pathologies (gastrointestinal, cardiopathic or asymptomatic). However, complement inhibition assays revealed that the response found was different, being lower in pooled sera with cardiac affections compared to the highest inhibitions found for peptides C4–C7 in sera with gastrointestinal pathologies. Previous studies using mice infected with bioluminescence parasites of the CL-Brener strain, revealed the gut as the main reservoir for the long-term persistence of *T. cruzi*, with the parasite being cleared or not detected in other tissues like the heart^49^. Additionally, quantification of samples from megaesophagus found that 100% of cases were positive for the presence of parasite DNA^50^. Assuming that gastrointestinal cases could be related to the persistence of focal *T. cruzi* populations in the gut, then the constant release of EVs by those populations might contribute to the higher antibody response found in the gastrointestinal cases analyzed. Furthermore, the highest complement inhibition corresponds to the peptide C7 which belongs to the C-term region of the MASP52 protein, that was previously characterized as part of the MASP protein repertoire expressed by the CL-Brener strain, which supports the preferential tropism of this strain to the gut as mentioned above^34^,^49^.

The significant, unspecific polyclonal response during the acute phase of Chagas disease and the subsequent control of the expansion of IgG^+^ B cells, which are highly activated by the Fas/FasL ligand, has been clearly demonstrated by various authors^7^,^8^,^51^. This polyclonal lymphocyte B activation induced by MASP proteins has been also demonstrated in peptide arrays in which different IgM and IgG recognition patterns seems to be an intrinsic characteristic to the hypervariable region of MASP proteins^30^. Our study shows that the MASP C-term region induced a stronger and earlier (2 weeks pi) response for the IgM than the IgG isotopes (Fig. 5), which demonstrates that a very rapid humoral response exists for IgM but an insufficient switching occurs to IgG during the development of the disease in Balb/c mice. Similarly, the C-term region of the SAPA antigen (which belongs to the TS family of *T. cruzi*), shows high IgM titers at the acute phase of the disease with a reduction on the antibody titers of 40% when disease progresses into its chronic phase^52^. The low switching capacity of B-cells responding to the exposed MASP C-term region together with prior polyclonal activation by peptides of hypervariable regions of the MASP family of proteins might be part of the parasite’s programmed response to avoid immune recognition.

In conclusion, our results demonstrate the presence of the MASP C-terminal region in EVs and the presence of humoral response against this region with a very fast IgM response in infected mice. Furthermore, our findings suggest that the antigenic cargo of EVs actively released by *T. cruzi* might act as a countermeasure against the immunological host defense and thus as a way to evade the humoral immune response.

## Additional Information

**How to cite this article**: De Pablos, L. M. *et al.* The C-terminal region of *Trypanosoma cruzi* MASPs is antigenic and secreted via exovesicles. *Sci. Rep.* **6**, 27293; doi: 10.1038/srep27293 (2016).

## Supplementary Material

Supplementary Information

Supplementary Dataset 1
