# Supplementary material for: The C-terminal region of Trypanosoma cruzi MASPs is antigenic and secreted via exovesicles
Source: Sci Rep. 2016 Jun 8;6:27293. doi: 10.1038/srep27293 (PMC4897614; doi:10.1038/srep27293)
Supplement: Supplementary Information [file srep27293-s1.doc]

**Supplementary Files of**

**Manuscript Title:** “The C-terminal region of *Trypanosoma cruzi* MASPs is antigenic and secreted via exovesicles”

**Authors:** Luis Miguel De Pablos, Isabel María Díaz Lozano, Maria Isabel Jercic, Markela Quizada, Maria José Giménez, Eva Calabuig, Ana Margarita Espino, Alejandro Gabriel Schijman, Inés Zulantay, Werner Apt, Antonio Osuna.

**Supplementary Table S1.** MASP C-term sequences obtained after sequencing 17 clones from the CL-Brener strain and 23 from PAN4 strain of *T.cruzi*. (CL: CL Brener; PAN 4 : PAN4 strain). Preferential GPI-modification site (omega-site) highlighted in red, alternative GPI-modification site (omega-site) highlighted in orange.
